# Opposing roles of deubiquitinases in the regulation of IRF7 transcriptional activity

**DOI:** 10.1128/mbio.02820-25

**Published:** 2026-06-09

**Authors:** Shumin Fan, Pracheta Sengupta, Karan Chawla, Manoj Veleeparambil, Ritu Chakravarti, Saurabh Chattopadhyay

**Affiliations:** 1 Department of Medical Microbiology and Immunology, University of Toledo College of Medicine and Life Sciences 89021, Toledo, Ohio, USA; 2 Department of Microbiology, Immunology and Molecular Genetics, University of Kentucky College of Medicine 12252, Lexington, Kentucky, USA; 3 Department of Internal Medicine, University of Kentucky College of Medicine 12252, Lexington, Kentucky, USA; The University of North Carolina at Chapel Hill, Chapel Hill, North Carolina, USA

**Keywords:** IFN regulatory factor 7, ubiquitination, deubiquitinases, K27 ubiquitination, OTUD5, USP2, innate immunity

## Abstract

Virus infection rapidly induces the production and secretion of interferons (IFNs), which amplify the antiviral responses in infected and neighboring uninfected cells. IFN regulatory factor 7 (IRF7), the “master transcription factor,” is pivotal in IFN induction, particularly in myeloid cells. Ubiquitination of IRF7 is essential for its transcriptional activation; however, the underlying molecular mechanisms remain poorly understood. We hypothesized that deubiquitinases (DUBs) act as endogenous regulators of IRF7 activity, and conducted a genetic screen using an siRNA library of human DUBs. The screen identified USP2 as a positive regulator and OTUD5 as a negative regulator of IRF7 activity. OTUD5, an inducible DUB, interacted with IRF7 and inhibited its K63-linked ubiquitination, thereby suppressing IRF7 activation. Conversely, USP2 promoted IRF7 activity by binding to IRF7 and removing K27-linked ubiquitin chains, which we found to be inhibitory. Specifically, K27-linked ubiquitination impeded phosphorylation of IRF7, a critical step for its activation. Collectively, our independent lines of investigation, coupled with genetic screens and mechanistic studies, uncovered USP2 and OTUD5 as novel modulators of IRF7 function, providing novel insights into the regulation of antiviral immunity.

**IMPORTANCE:**

IFN regulatory factor 7 (IRF7) is a central protein that launches type I interferon responses, and its timely activation is essential for antiviral immunity. Our study uncovers a mechanism by which IRF7 activation is controlled by enzymes that specifically remove small molecular tags, known as ubiquitin, from proteins. Through a focused screen, we identified two enzymes with opposing roles in modulating IRF7 activity. OTUD5, one of these enzymes, suppresses IRF7 activity by removing a ubiquitin tag that is essential for its transcriptional function. In contrast, USP2, the other enzyme, activates IRF7 by removing a ubiquitin tag that is inhibitory to IRF7 functions. These findings reveal previously unrecognized layers of IRF7 regulation and highlight how these enzymes can be targeted therapeutically in diseases driven by abnormal IRF7 functions.

## INTRODUCTION

The type I interferon (IFN) system represents the first line of defense against viral infections. Type I IFNs, a class of cytokines, primarily produced and secreted in response to viral infection, play a central role in initiating and shaping both innate and adaptive immune responses (1, 2). IFN production is triggered by the recognition of pathogen-associated molecular patterns and damage-associated molecular patterns by pattern recognition receptors (PRRs). Activated PRRs initiate intracellular signaling cascades that converge on interferon regulatory factors (IRFs), a family of transcription factors critical for the induction of IFNs and other inflammatory genes (3–5). Among the nine IRF family members identified in mammals, IRF1, IRF3, IRF5, IRF7, and IRF8 function as positive regulators of PRR-induced type I IFN responses (6). IRF3 and IRF7 are structurally related and serve as key transcription factors for type I IFN production downstream of cytosolic RNA sensors (RIG-I-like receptors), DNA sensors (cGAS), and endosomal Toll-like receptors (TLRs). IRF5, while also important for inducing type I IFNs, plays a critical role in driving the expression of proinflammatory cytokines such as IL-6, TNF-α, and IL-12. Interplay among IRF3, IRF5, and IRF7, therefore, is essential for determining the shape and the outcome of the innate immune response (5, 6).

Dysregulation of IRFs is associated with pathological conditions, including autoimmune disorders, cancer, and metabolic diseases. In autoimmune diseases such as systemic lupus erythematosus and rheumatoid arthritis, aberrant IRF activity causes excessive IFN production, contributing to chronic inflammation and tissue damage. In cancer, IRFs may either support anti-tumor immunity or facilitate immune evasion and tumor progression, e.g., IRF1 and IRF7 have been shown to suppress tumor metastasis. In metabolic disorders, IRF-mediated inflammation may exacerbate insulin resistance and metabolic dysfunction (5, 7). These findings highlight the importance of tightly regulated IRF activity in maintaining immune homeostasis and underscore the potential of IRFs as therapeutic targets.

IRF7 is considered the “master regulator” of type I IFN responses, particularly in immune cells, and plays a pivotal role in the amplification of IFN signaling. While IRF7 expression is inducible by IFNs and PRR signaling, its transcriptional activity depends on posttranslational modifications, primarily phosphorylation. Upon activation by PRRs, adaptor proteins such as MyD88, TRIF, MAVS, and STING recruit the kinases IKKε and TBK1, which phosphorylate IRF7 at key serine residues (e.g., Ser477 and Ser479). This phosphorylation facilitates IRF7 dimerization and nuclear translocation, enabling the transcription of IFN-α and other ISGs (8). In addition to phosphorylation, ubiquitination serves as a critical regulatory mechanism of IRF7 activity. Ubiquitin (Ub), a 76-amino acid protein, can form polyubiquitin chains through any of its seven lysine residues (K6, K11, K27, K29, K33, K48, and K63), with distinct chain types imparting different functional outcomes on target proteins (9–11). For example, K48-linked ubiquitination commonly targets proteins for proteasomal degradation, whereas K63-linked ubiquitination can enhance protein activation and signaling. In Epstein-Barr virus (EBV) infection, the viral protein LMP1 promotes IRF7 activation via K63-linked ubiquitination at Lys444, Lys446, and Lys452, mediated by the E3 ligase TRAF6. This modification facilitates the recruitment of IKKε, linking IRF7 ubiquitination to its phosphorylation and activation (11–15).

Excessive accumulation of K63-linked ubiquitinated IRF7 (Ub^63^-IRF7), however, can lead to sustained IFN production, contributing to immunopathology and autoimmunity. Therefore, deubiquitination plays a vital regulatory role in modulating IRF7 activity and maintaining immune balance. Deubiquitinases (DUBs) are a class of enzymes that remove ubiquitin chains from proteins, thereby regulating protein turnover, signaling activity, and functional outcomes. DUBs can either enhance or suppress the activity of their targets by selectively cleaving specific ubiquitin linkages (16, 17). They are broadly classified into four families: ubiquitin-specific proteases (USPs), ubiquitin C-terminal hydrolases, ovarian tumor proteases (OTUs), and JAMM metalloproteases (18). DUBs are also essential for maintaining the ubiquitin-proteasome system and ensuring proper recycling of free ubiquitin (19). In EBV infection, viral protein LMP1 recruits the DUB A20 to remove K63-linked ubiquitin chains from IRF7, thereby dampening IRF7 activation and IFN responses (20).

Despite their importance, systematic studies to identify specific roles of DUBs in regulating mammalian IRF7 remain poorly understood. In this study, we sought to identify DUBs that modulate IRF7 activity by performing an siRNA-based screen targeting human DUBs. Our screen revealed two novel IRF7 regulators—USP2, an activator DUB (DUB^A^), and OTUD5, an inhibitor (DUB^I^). Mechanistically, USP2 enhances IRF7 activity by removing inhibitory K27-linked ubiquitin chains, whereas OTUD5 attenuates IRF7 function by cleaving activating K63-linked chains. These findings provide new insights into the posttranslational regulation of IRF7 and offer potential therapeutic targets for modulating IFN responses in viral and inflammatory diseases.

## MATERIALS AND METHODS

### Antibodies

The antibodies used in the studies are anti-actin (Sigma-Aldrich #A5441), anti-FLAG (Cell Signaling Technology [CST] #2368 and Sigma-Aldrich #F1804), anti-HA (CST #3724S), anti-HDAC1 (CST #34589), anti-IRF3 and anti-IFIT (as previously described [21–23]), anti-IRF7 (CST #39659 and Santa Cruz Biotechnology #sc-74471), anti-phospho-IRF7 (CST #24129), anti-OTUD5 (Invitrogen #PA5-20611), anti-TRAF6 (Santa Cruz Biotechnology #sc-7221), anti-USP2 (Proteintech #10392-1-AP and Invitrogen #MA5-37724), and anti-ubiquitin (CST #58395S), and anti-V5 (CST #13202).

### Cell lines, plasmids, and viruses

HEK293T/17 (CRL-11268) and RAW264.7 (TIB-71) cells were obtained from ATCC. RAW-Lucia ISG (RAWL-ISG) cells were from Invivogen. Cells were cultured in DMEM supplemented with 10% fetal bovine serum and 1% penicillin/streptomycin at 37°C in 5% CO_2_. Mycoplasma removal agent (MP Biomedicals) was routinely used. Plasmids included HA-Ub-K27O (Addgene #22902), HA-Ub-K63O (Addgene #17606), OTUD5 (Sino Biologicals #HG22452-CF), and USP2 (Sino Biologicals #HG12713-UT). Sendai virus (SeV Cantell) was from Charles River Laboratories. GFP-expressing VSV (GFP.VSV) and IAV strain PR8 were previously described (23, 24). SeV and vesicular stomatitis virus (VSV) stocks were quantified using plaque assay (pfu/mL), and IAV stocks were quantified using TCID_50_/mL.

### Gene knockout and overexpression cell lines

IRF3 knockout (KO) HEK293T cells (HEK-3KO) were generated using CRISPR/Cas9 as described (22). V5-tagged IRF7 was used to generate HEK-3KO.IRF7 stable cell lines. Knockout and overexpression were confirmed by immunoblotting.

### Cloning and mutagenesis

Mouse IRF7 (Origene: NM_016850) was subcloned into the pLVX-IRES-puromycin vector (Clontech) to generate V5-tagged wild-type and deletion mutants (22). Constructs were generated using standard molecular biology techniques and verified by sequencing. Lysine-to-arginine (KR) point mutants of IRF7 were synthesized by GenScript (Fig. S1).

### Cell lysis and immunoblotting

Cells were lysed in 50 mM Tris-HCl (pH 7.4), 150 mM NaCl, 0.1% Triton X-100, 1 mM sodium orthovanadate, 10 mM NaF, 10 mM β-glycerophosphate, 5 mM sodium pyrophosphate, and protease/phosphatase inhibitors. Lysates were sonicated and centrifuged (30 min), and protein concentrations were measured using Bradford reagent (Bio-Rad #500-0006). Equal amounts of protein were analyzed via SDS-PAGE and immunoblotting.

### Co-immunoprecipitation and ubiquitination assays

For co-immunoprecipitation (co-IP), cells were lysed in EPPS buffer with protease/phosphatase inhibitors (Roche), subjected to freeze-thaw cycles, and centrifuged. For ubiquitination assays, cells were lysed in Tris buffer (50 mM, pH 7.4) with 150 mM NaCl, 0.2% Triton X-100, 0.5% SDS, 1 mM sodium orthovanadate, 10 mM NaF, 10 mM β-glycerophosphate, 5 mM sodium pyrophosphate, protease/phosphatase inhibitors (Roche), and MG132. Lysates were subjected to freeze-thaw cycles and centrifuged at 13,000 rpm. Immunoprecipitation was performed using V5-agarose beads (Sigma #SAE0203) overnight, followed by sequential washing with EPPS and RIPA buffers. Bound proteins were eluted by boiling in SDS buffer with 2-mercaptoethanol and analyzed by SDS-PAGE and immunoblotting. Nuclear fractions were isolated as previously described (22, 23).

### Cellular signaling and viral infections

TLR3 signaling was induced by adding polyI:C (pIC, 25 µg/mL) to the culture medium for 8 h. retinoic acid inducible gebe I (RIG-I) signaling was activated by transfecting cells with pIC using Lipofectamine 2000 (Thermo Fisher Scientific), denoted as pIC + LF, for 8 h unless otherwise noted. TLR9 signaling was activated by adding CpG ODN (10 µg/mL) to the culture media (25, 26), and TLR7 signaling was stimulated by adding R848 (10 µg/mL) to the culture media (27). For virus infections, cells were adsorbed with SeV (MOI 10), IAV (MOI 5), or GFP.VSV (MOI 0.1) in serum-free DMEM for 2 h, then washed and incubated in complete medium. Cells were harvested at the indicated time points for analysis by qRT-PCR or immunoblotting.

### Proximity ligation assay and confocal microscopy

Cells grown on coverslips were fixed with 4% paraformaldehyde (Electron Microscopy Sciences #15710) and permeabilized with 0.2% Triton X-100 (Thermo Fisher Scientific #9002-93-1). Cells were immunostained with anti-IRF7 and anti-OTUD5 or anti-USP2 antibodies, followed by Duolink proximity ligation assay (PLA; Sigma-Aldrich: DUO92008-3, DUO92004, and DUO92002) per the manufacturer’s instructions. Coverslips were mounted using VectaShield with DAPI (Vector Laboratories #H-1200) and imaged using a Zeiss microscope (Zen Blue) and Zeiss Zen 3.7 software.

### siRNA transfection

The siRNAs targeting human TRAF6, OTUD5, USP2, and a SMARTpool siRNA library for human deubiquitinating enzymes (10 μM, Horizon Discovery #G-104705, Lot 19124) were transfected using DharmaFECT-1 reagent (Horizon Discovery) in HEK cells and DharmaFECT-3 in RAW264.7 and BMDMs. Murine Otud5 (#L-013823-00) and Usp2 (#L-006069-00) specific siRNAs used in the study were obtained from Horizon Discovery. A non-targeting siRNA (Horizon Discovery #D-001810-10-05) was used as a control. After 48 h, cells were used for co-IP, ubiquitination, or qRT-PCR analyses, as described in the figure legends.

### Primary cell culture

Bone marrow-derived macrophages (BMDMs) were isolated from wild-type male and female mice using established protocols (25, 28).

### DUB screening protocol for IRF7 regulation

HEK-3KO.IRF7 cells were transfected with control (siNT) or DUB-specific siRNAs (siDUBs) for 60 h. Cells were infected with SeV (MOI 10) for 8 h, and IFIT3 expression was analyzed by immunoblot. Twenty-four candidate DUBs shortlisted from the primary screen using arbitrary thresholds (>1.5- and <0.5-fold IFIT3 protein expression, for inhibitor and activator DUBs, respectively) were further tested in secondary screening via qRT-PCR for *IFNB1* mRNA expression. Fourteen DUBs were selected for tertiary screening, in which immunoprecipitation and qRT-PCR analyses were used to assess IRF7 ubiquitination and *IFIT1* mRNA induction, respectively. An overview of the screening workflow is presented in Fig. 2A, and results from these screens are summarized in Fig. 3F.

### RNA isolation and qRT-PCR

RNA was extracted using TRIzol (Sigma #T9424) and treated with DNase I (Promega). Synthesis of cDNA was performed using random hexamers and the ImProm-II Reverse Transcription Kit (Promega). qRT-PCR was carried out using Radiant SYBR Green PCR mix (Alkali Scientific Inc.) on Roche LightCycler 96 and Applied Biosystems 7500 Fast Dx instruments. Data were analyzed using LightCycler 480 v1.5 and SDS v1.4.1 software. Expression levels were normalized to 18S rRNA and plotted using GraphPad Prism 10. Primer sequences are listed in Fig. S2.

### Quantification and statistical analysis

The data represent at least three independent experiments, and statistical analyses were conducted using GraphPad Prism 10, incorporating both biological and technical replicates. Two-tailed unpaired Student’s *t*-tests were used for two-group comparisons and one- or two-way ANOVA for multiple-group comparisons. *P*-values < 0.05 were considered statistically significant and are presented in the figure legends.

## RESULTS

### Establishing a human cell model to screen regulators of IRF7 transcriptional activity

**Fig 1:**
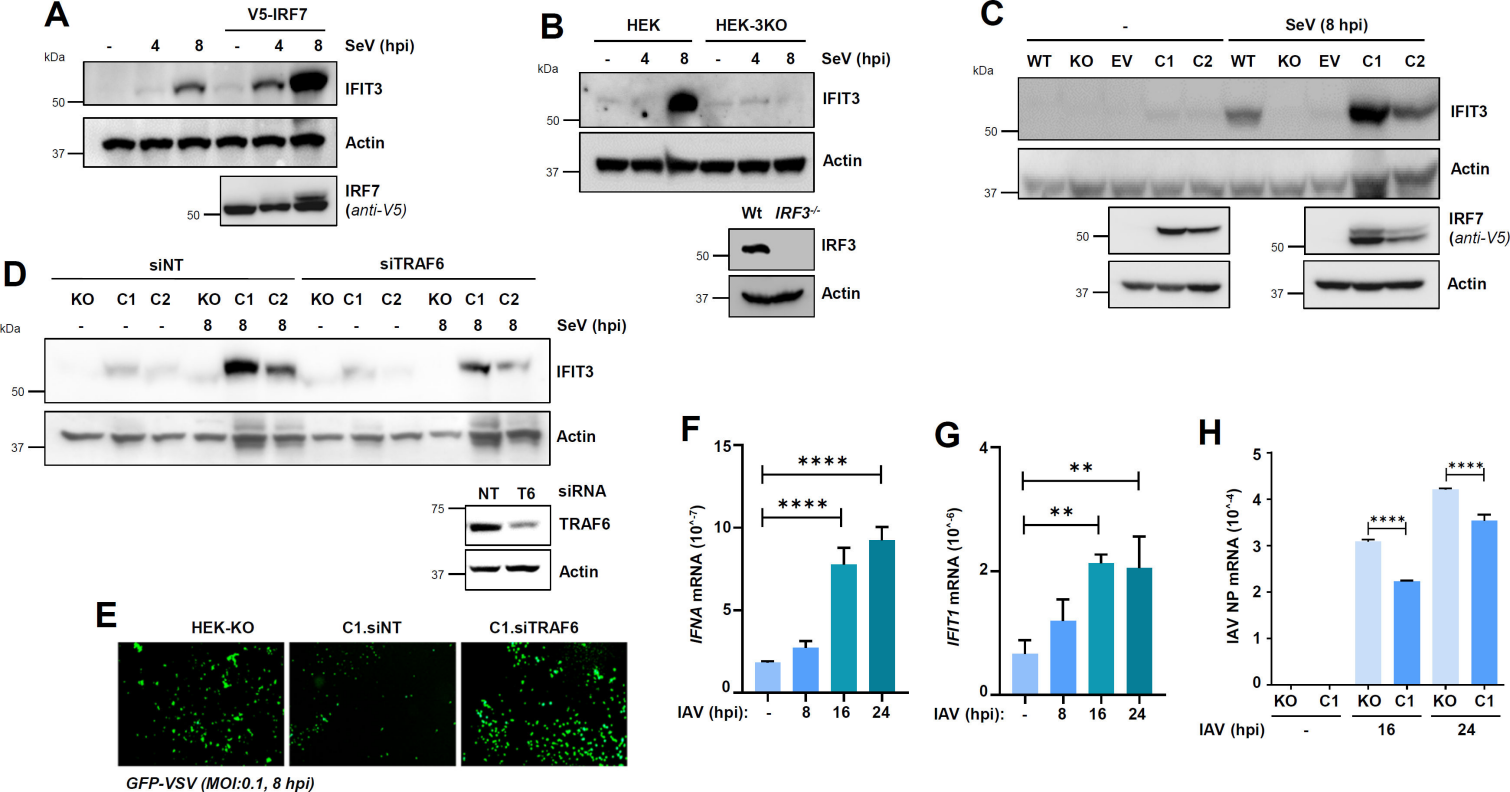
Establishing a functional screening model for IRF7-specific regulators in the absence of IRF3. (**A**) HEK293T (HEK) cells were transfected with V5-tagged IRF7 and infected with SeV for the indicated times. IFIT3 and IRF7 expressions were analyzed by immunoblotting. (**B**) Wild-type (Wt) and IRF3-deficient (HEK-3KO) HEK293T cells (HEK) were infected with SeV as indicated, and IFIT3 induction was assessed by immunoblot. IRF3 protein levels are shown in the lower panel. (**C**) HEK-3KO cells were transfected with V5.IRF7, and two stable clones (C1 and C2) were tested for IFIT3 induction upon SeV infection. IRF7 expression is shown in the lower panels. (**D**) Clones C1 and C2 were transfected with NT or TRAF6-specific siRNAs and analyzed for IFIT3 induction upon SeV infection by immunoblot. TRAF6 knockdown is shown below. (**E**) HEK-3KO and HEK-3KO.IRF7 (C1) cells were transfected with NT or TRAF6-specific siRNAs and infected with GFP.VSV. Representative fluorescence microscopy images are shown. (**F–H**) HEK-3KO.IRF7 cells were infected with influenza A virus (IAV; MOI: 5) for the indicated times. *IFNA*, *IFIT1*, and viral NP mRNA levels were quantified by qRT-PCR. EV, empty vector; NT, non-targeting. The data represent mean ± SEM (**F–H**); **P < 0.001 and ****P < 0.0001.

Early antiviral responses are primarily mediated by IRF3, which is ubiquitously expressed and rapidly activated upon infection. However, many viruses target IRF3 for degradation to evade host immune defenses. To investigate whether IRF7 can functionally compensate for IRF3, we established a HEK293T (HEK) cell model with ectopic IRF7 expression to mimic its physiological induction during infection. Compared to parental cells, IRF7-expressing cells showed increased induction of the antiviral gene IFIT3 following SeV infection (Fig. 1A), indicating that IRF7 is transcriptionally functional in this model. To assess IRF7’s ability to replace IRF3, we generated an IRF3 knockout HEK cell line (HEK-3KO) using CRISPR/Cas9, which, as expected, failed to induce IFIT3 upon SeV infection (Fig. 1B). We then ectopically expressed V5-tagged IRF7 (V5.IRF7) in HEK-3KO cells and isolated two clones (C1 and C2) with different IRF7 expression levels. Both clones induced IFIT3 upon SeV infection in the absence of IRF3 (Fig. 1C), indicating that IRF7 can replace IRF3 for antiviral gene induction. To determine whether IRF7 activation depended on canonical antiviral signaling in these cells in the absence of IRF3, we used siRNA to knock down TRAF6, a known E3 ubiquitin ligase for IRF7. TRAF6 knockdown, as expected, reduced SeV-induced IFIT3 expression in both clones (Fig. 1D). The impaired antiviral gene expression correlated with enhanced replication of VSV, as indicated by increased GFP reporter expression (Fig. 1E). We selected clone C1 (HEK-3KO.IRF7) for further analysis using influenza A virus (IAV) infection. Compared to HEK-3KO cells, HEK-3KO.IRF7 cells upregulated IRF7-target genes (*IFNA* and *IFIT1*; Fig. 1F and G) and suppressed IAV NP mRNA levels (Fig. 1H), confirming antiviral activity. Together, these data establish HEK-3KO.IRF7 as a functional human cell model in which IRF7 can substitute for IRF3 to drive antiviral gene expression and restrict viral replication. This model, therefore, provides a valuable platform to screen for specific regulators of IRF7 transcriptional activity.

### A genetic screen to identify deubiquitinases regulating IRF7 transcriptional activity

**Fig 2:**
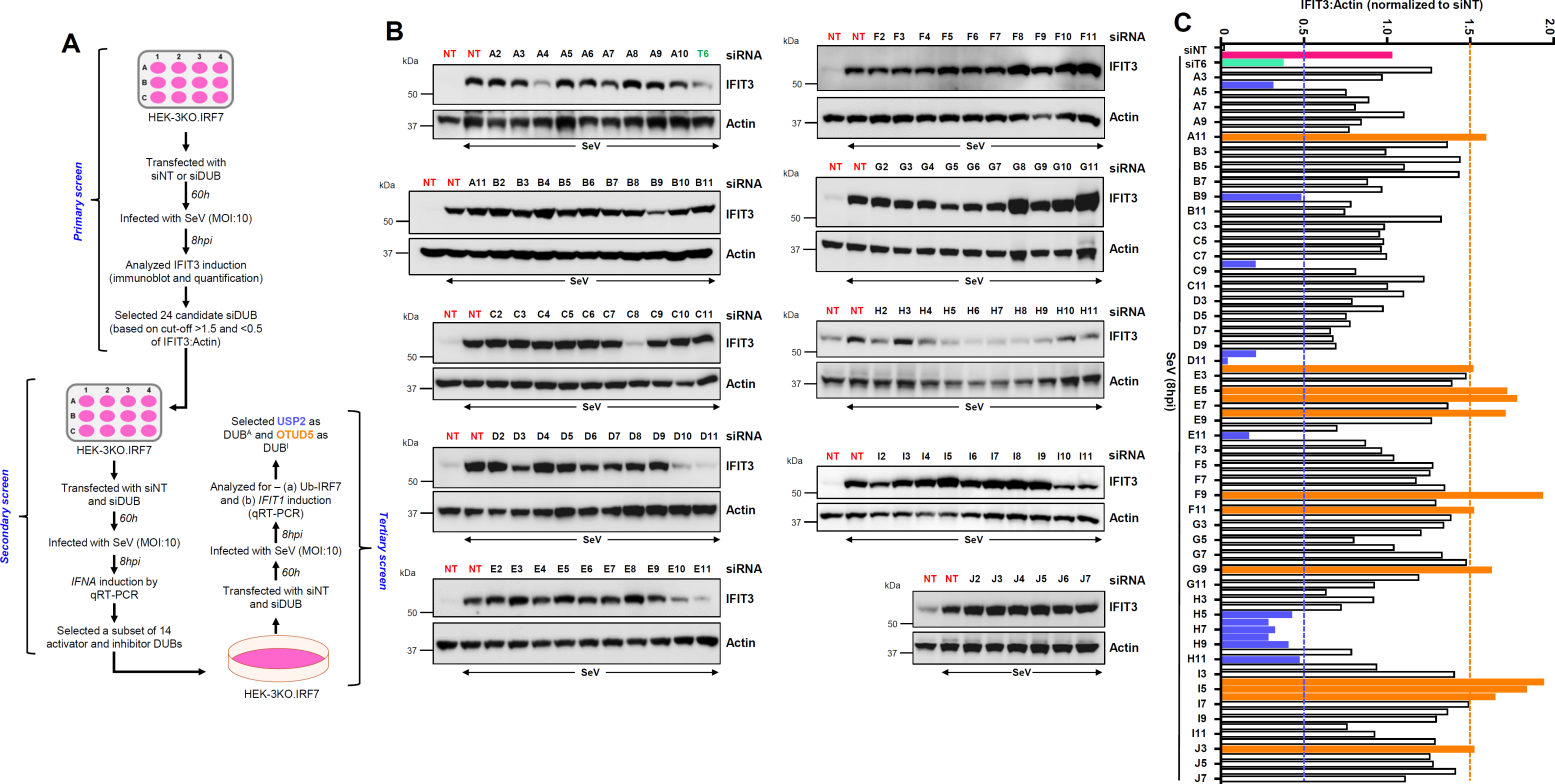
Primary siRNA screen of human deubiquitinases (DUBs) to identify IRF7 regulators. (**A**) Schematic overview of the DUB siRNA screening workflow in HEK-3KO.IRF7 cells. Cells were transfected with siRNAs, infected with SeV, and analyzed for IFIT3 induction by immunoblot (primary screen). The shortlisted candidates from the primary screen were followed by secondary and tertiary screens, as indicated. (**B**) HEK-3KO.IRF7 cells were transfected with individual DUB siRNAs (10 µM) for 60 h, followed by SeV infection (MOI:10) for 8 h. IFIT3 expression was analyzed by immunoblot. TRAF6 siRNA served as a positive control; NT siRNA was included in each batch. (**C**) Densitometric quantification of IFIT3 induction from panel **B**, normalized to actin using ImageJ. The broken lines indicate the arbitrary thresholds used to shortlist the activators (<0.5-fold) and inhibitors (>1.5-fold). NT, non-targeting.

The specific Ub linkages conjugated to IRF7, and the DUBs responsible for their removal, remain poorly understood. To identify novel DUBs that regulate IRF7 transcriptional activity, we employed the HEK-3KO.IRF7 human cell model to conduct a genetic screen using an siRNA library against human DUBs (Fig. 2A). In the primary screen, HEK-3KO.IRF7 cells were transfected with pooled siRNAs (four per gene) targeting individual human DUBs, alongside a non-targeting control (siNT). Following siRNA transfection, cells were infected with SeV, and IRF7-mediated induction of the antiviral protein IFIT3 was assessed by immunoblotting (Fig. 2B). IFIT3 expression was normalized to actin, and relative levels were quantified against siNT controls (Fig. 2C). As a positive control, siRNA against TRAF6 (siT6), a known upstream activator of IRF7, effectively suppressed IFIT3 induction, validating the screen.

**Fig 3:**
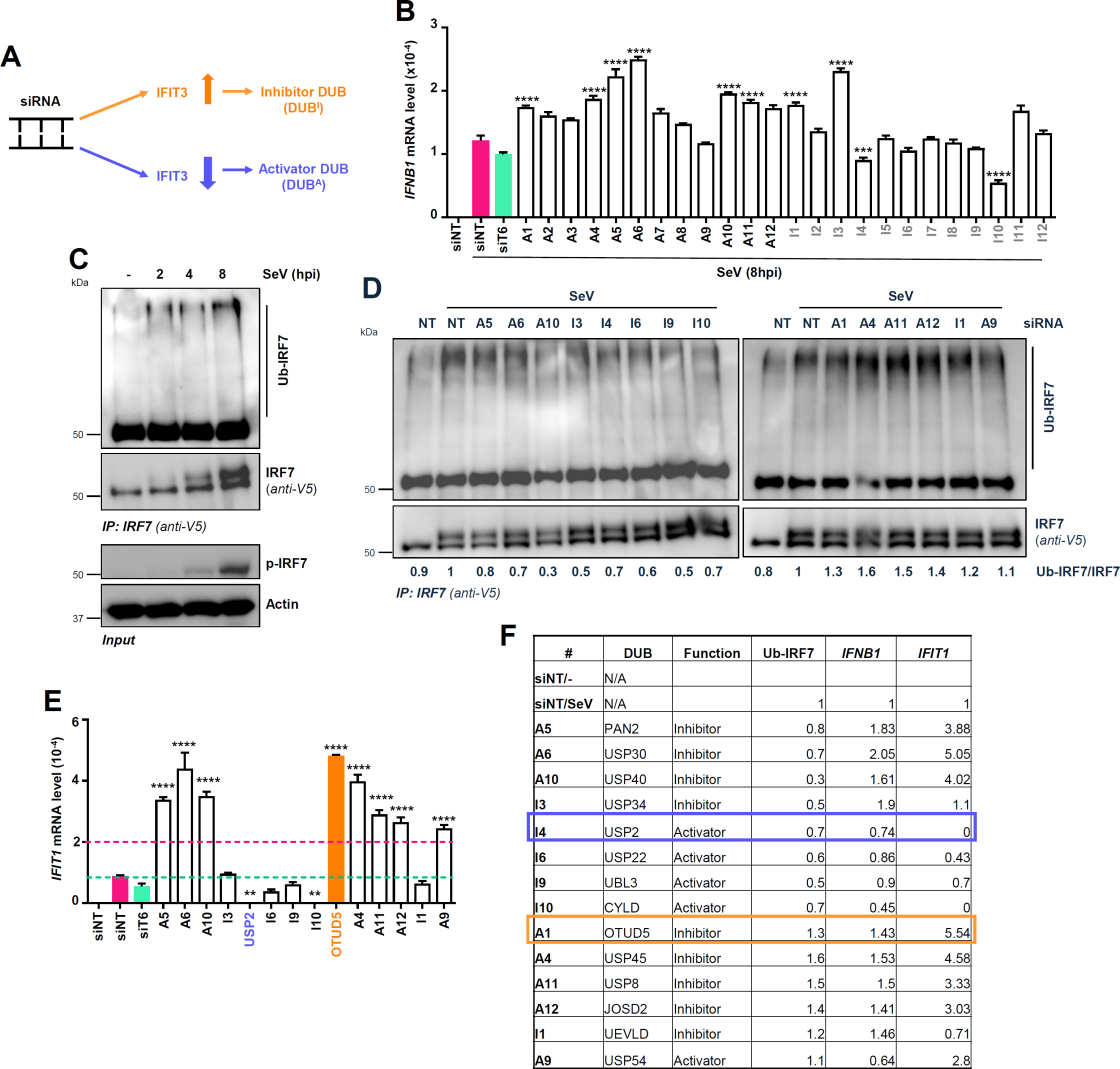
Secondary and tertiary screening identify USP2 and OTUD5 as IRF7-specific regulators. (**A**) Criteria used to select candidate DUBs for follow-up screening. (**B**) In secondary screening, selected DUB siRNAs were transfected into HEK-3KO.IRF7 cells, and *IFNB1* mRNA levels were measured by qRT-PCR after SeV infection. (**C**) HEK-3KO.IRF7 cells were infected with SeV for the indicated times. IRF7 ubiquitination was analyzed by immunoprecipitation using anti-V5 antibody, followed by anti-ubiquitin (Ub) immunoblot. Input cell lysates were probed for p-IRF7. (**D**) Candidate DUB siRNAs from panel **B** were tested for IRF7 ubiquitination using the assay in panel **C**, and relative Ub-IRF7 levels were quantified by Image J. (**E**) *IFIT1* mRNA induction in response to SeV infection was quantified by qRT-PCR in cells transfected with candidate DUB siRNAs. (**F**) Summary of secondary and tertiary screen results. Highlighted rows indicate DUB candidates selected for further study. NT, non-targeting.

Contrary to our expectations, the primary screen revealed both potential positive and negative DUB regulators of IRF7 activity, based on IFIT3 protein levels relative to Actin (Fig. 2C). We reasoned that knockdown of an activating DUB would reduce IFIT3 expression, whereas knockdown of an inhibitory DUB would enhance it (Fig. 3A). Using an arbitrary threshold (fold change > 1.5 for inhibitors and < 0.5 for activators), we shortlisted 12 candidate activator DUBs and 12 inhibitor DUBs for secondary screening (Fig. S3). In the secondary screen, we assessed IRF7-dependent *IFNB1* mRNA expression by qRT-PCR in response to SeV infection. Quantification of *IFNB1* mRNA further enabled us to exclude the role of these DUBs in regulating IFIT protein turnover via the ubiquitination machinery. This analysis allowed us to narrow the list to seven candidate activators and seven candidate inhibitors (Fig. 3B). We observed that several activators and inhibitors identified by the primary screen exerted opposing effects on *IFNB1* mRNA expression, a criterion we used to prioritize candidates for subsequent analyses. These 14 DUBs were advanced to a tertiary screen assessing IRF7 ubiquitination—the most proximal readouts of their effects on the IRF7 substrate—as well as induction of a second IRF7 target gene, *IFIT1*. To analyze steady-state ubiquitination of IRF7, we developed a biochemical assay to detect ubiquitinated IRF7 (Ub-IRF7) under native conditions, without protease inhibitors, to reflect physiological turnover. SeV infection robustly induced Ub-IRF7 (Fig. 3C). IRF7 phosphorylation was also detected by a mobility shift using anti-V5 antibody, which was confirmed using phospho-serine (pSer)-specific antibody (Fig. 3C, bottom panel). Each of the fourteen DUB candidates was individually knocked down by siRNA, and Ub-IRF7 levels were quantified (Fig. 3D), and *IFIT1* mRNA induction was measured by qRT-PCR (Fig. 3E). Results from the secondary and tertiary screens were integrated to compare candidate DUBs based on four criteria: (i) regulation of Ub-IRF7 levels, (ii) impact on *IFNB1* mRNA expression, (iii) effect on *IFIT1* mRNA induction, and (iv) novel regulators of interferon responses (Fig. 3F). From this analysis, we identified USP2 as a candidate activator DUB (DUB^A^) and OTUD5 as a candidate inhibitor DUB (DUB^I^) of IRF7 transcriptional activity (Fig. 3F, boxed) and selected them for further mechanistic studies.

### USP2 functions as a positive regulator and OTUD5 as a negative regulator of IRF7 transcriptional activity

**Fig 4:**
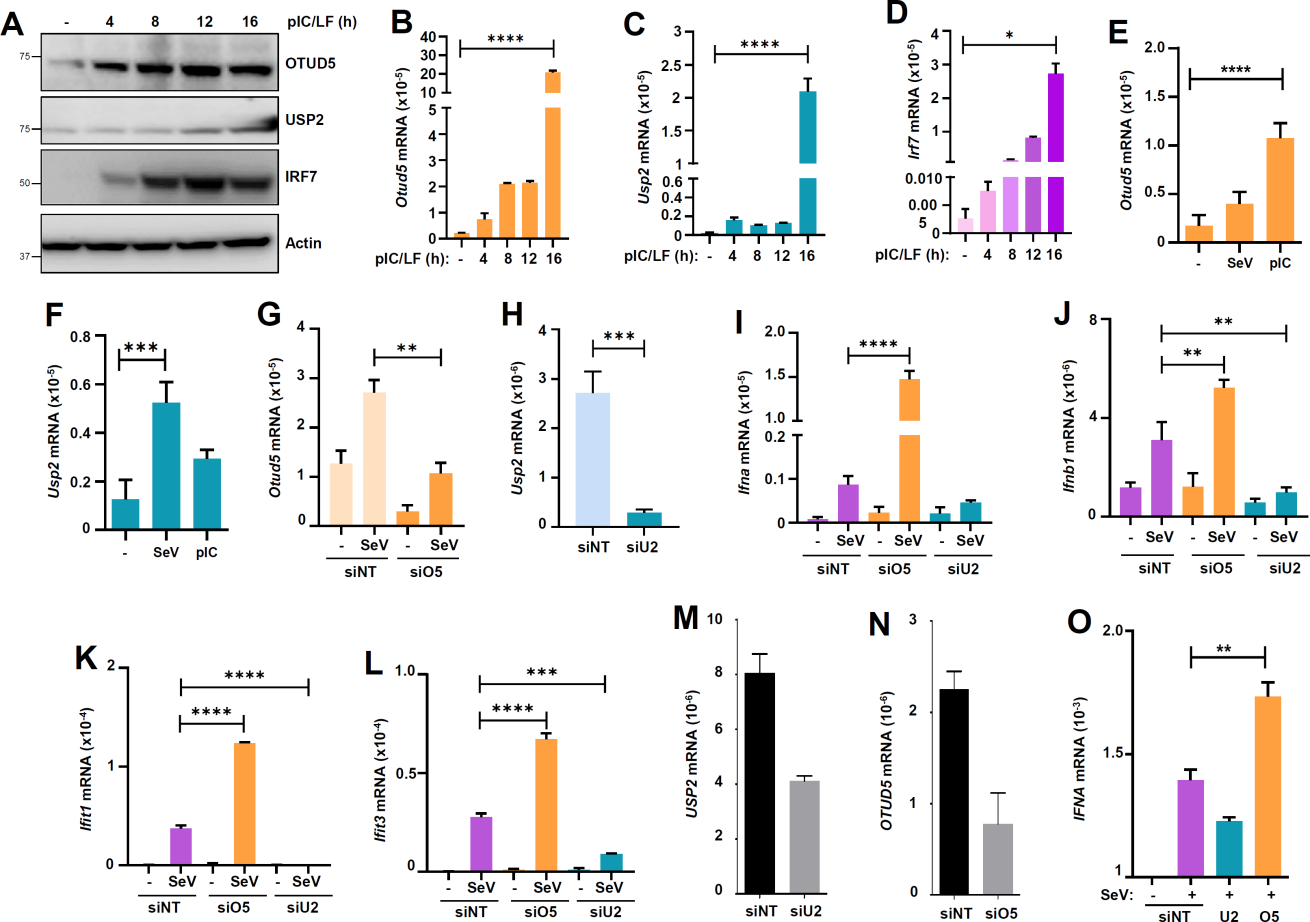
USP2 and OTUD5 differentially regulate IRF7 activity in myeloid and non-myeloid cells. (**A**) RAW264.7 cells were transfected with polyI:C (pIC/LF) for the indicated time, and the cell lysates were analyzed for OTUD5, USP2, and IRF7 using immunoblot. (**B–D**) RAW264.7 cells were transfected with polyI:C (pIC/LF) for the indicated time when *Otud5*, *Usp2*, and *Irf7* mRNA levels were analyzed by qRT-PCR. (**E and F**) RAW-Luci macrophages were either infected with SeV or treated with pIC. *Otud5* and Usp2 mRNA levels were quantified by qRT-PCR. (**G–L**) RAW264.7 cells were transfected with NT, Otud5 (O5), or Usp2 (U2) siRNAs, infected with SeV for 8 h, and mRNA levels of *Otud5, Usp2*, *Ifna*, *Ifnb1*, *Ifit1*, and *Ifit3* were analyzed by qRT-PCR. (**M–O**) HEK-3KO.IRF7 cells were transfected with NT, OTUD5 (O5), or USP2 (U2) siRNAs and infected with SeV. *USP2*, *OTUD5*, and *IFNA* mRNA levels were measured by qRT-PCR. The data represent mean ± SEM (**A–G and J**), *P < 0.05, **P < 0.001, ***P < 0.001, and ****P < 0.0001.

To elucidate the mechanisms by which USP2 and OTUD5 regulate IRF7 transcriptional activity, we used RAW264.7 macrophages, a murine immune cell model, in which RLR stimulation caused robust upregulation of OTUD5 and USP2, and as expected, IRF7 proteins (Fig. 4A). To investigate whether *Otud5* and *Usp2* genes were transcriptionally upregulated, we performed qRT-PCR analyses, which showed strong induction of *Otud5*, *Usp2*, and *Irf7* mRNAs upon RLR stimulation (Fig. 4B through D). Similar upregulation of *Otud5* and *Usp2* was also observed in SeV-infected and TLR3-stimulated cells (Fig. 4E and F). To assess the functional relevance of OTUD5 and USP2 in these cells, we performed siRNA-mediated knockdown (Fig. 4G and H). As expected from our screen results, knockdown of *Otud5* significantly enhanced the expression of IRF7-dependent antiviral genes—*Ifna*, *Ifnb1*, *Ifit1*, and *Ifit3*, compared to non-targeting control siRNA (Fig. 4I through L). Conversely, knockdown of *Usp2* led to a marked reduction in the expression of the same set of genes (Fig. 4I through L), supporting the conclusion that USP2 functions as a positive regulator (DUB^A^) and OTUD5 as a negative regulator (DUB^I^) of IRF7 activity. To confirm that these effects were independent of IRF3, which is also expressed in RAW264.7 cells, we validated the findings in the HEK-3KO.IRF7 cell model that lacks IRF3. In these cells, knockdown of USP2 suppressed, while knockdown of OTUD5 enhanced, *IFNA* expression following viral stimulation (Fig. 4M through O), consistent with the observations in macrophages. Together, these results confirm that USP2 promotes and OTUD5 suppresses IRF7 transcriptional activity, validating their roles as DUB^A^ and DUB^I^, respectively, in both human and murine cellular contexts.

### USP2 and OTUD5 interact with IRF7 during RLR stimulation and viral infection

**Fig 5:**
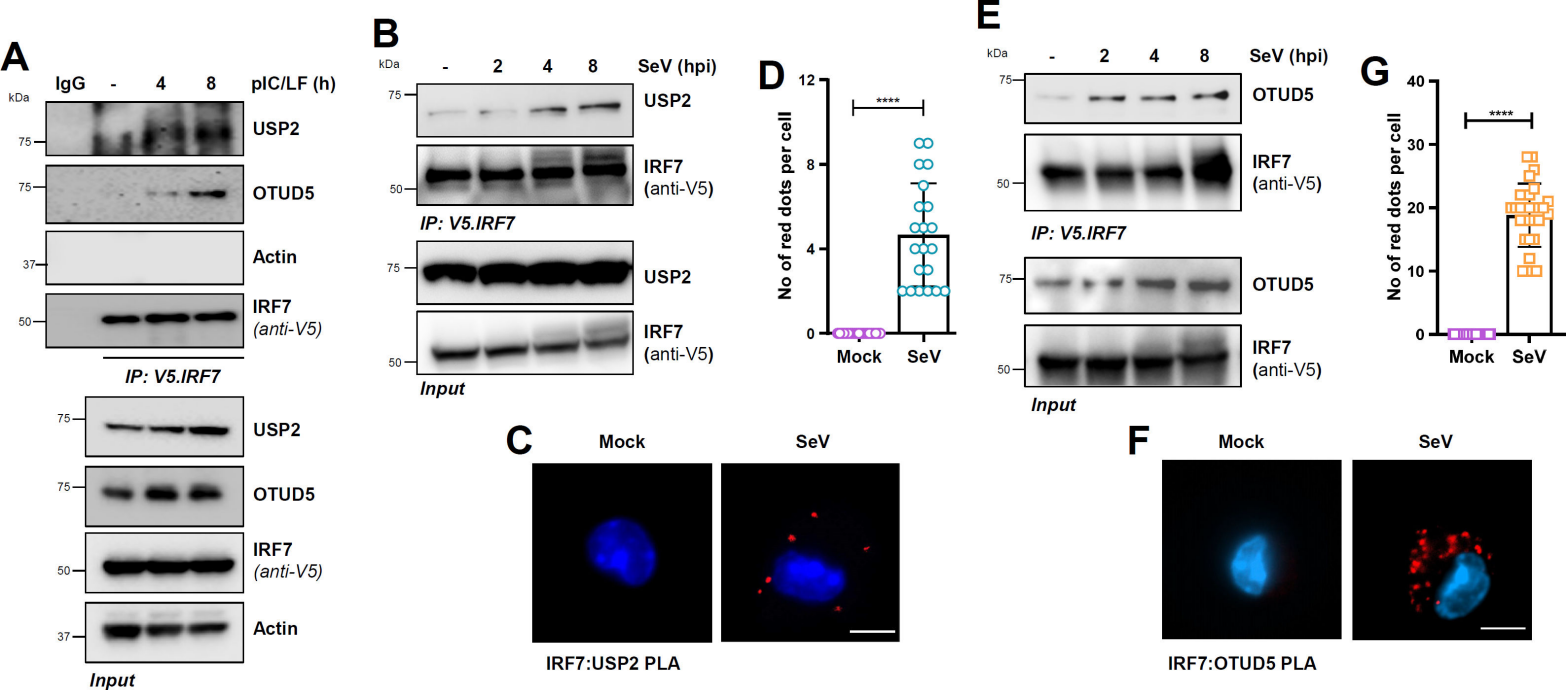
IRF7 interacts with USP2 and OTUD5 upon RLR stimulation and SeV infection. (**A**) HEK-3KO.IRF7 cells were transfected with polyI:C (pIC/LF) for the indicated times, and cell lysates were immunoprecipitated with anti-V5 (IRF7), and the immunoprecipitates were analyzed by immunoblot for USP2, OTUD5, and actin. (**B**) USP2:IRF7 interaction in HEK-3KO.IRF7 cells was assessed by co-immunoprecipitation at the indicated times post-SeV infection. (**C and D**) Proximity ligation assay (PLA) was performed in Wt primary BMDMs using anti-USP2 and anti-IRF7 antibodies in mock or SeV-infected (8 hpi) cells. Red dots represent interaction signals (**C**), quantified in panel **D**. (**E**) OTUD5:IRF7 interaction in HEK-K3O.IRF7 cells was analyzed by co-immunoprecipitation following SeV infection. (**F and G**) PLA was conducted in Wt BMDMs using anti-OTUD5 and anti-IRF7 antibodies in mock or SeV-infected cells. Interaction signals (**F**) were quantified in panel **G**.

Given their roles as regulators of IRF7 transcriptional activity, we investigated whether USP2 and OTUD5 interact with IRF7 during antiviral responses. Using HEK-3KO.IRF7 cells, we performed co-IP to assess the interaction of endogenous USP2 and OTUD5 with IRF7. Both USP2 and OTUD5 showed robust stimulation-dependent interaction with IRF7 in RLR-activated cells (Fig. 5A). Next, we examined whether these proteins interact with IRF7 in virus-infected cells. Indeed, USP2 showed a low basal association with IRF7 in uninfected cells, which markedly increased upon SeV infection (Fig. 5B). To validate this interaction in primary immune cells, we performed PLAs in primary BMDMs. SeV infection led to a robust increase in PLA signal for USP2:IRF7 interaction, confirming SeV-induced complex formation in a physiologically relevant model (Fig. 5C and D; Fig. S4). We next examined the interaction between IRF7 and OTUD5. Similar to USP2, endogenous OTUD5 co-immunoprecipitated with IRF7 in HEK-3KO.IRF7 cells during SeV infection (Fig. 5E). PLA analysis in SeV-infected BMDMs further confirmed a robust increase in IRF7-OTUD5 interaction (Fig. 5F and G; Fig. S4). These findings indicate that both USP2 and OTUD5 interact with IRF7 in response to viral infection and RLR stimulation in myeloid and non-myeloid cells.

### USP2 and OTUD5 regulate IRF7 through linkage-specific deubiquitination

**Fig 6:**
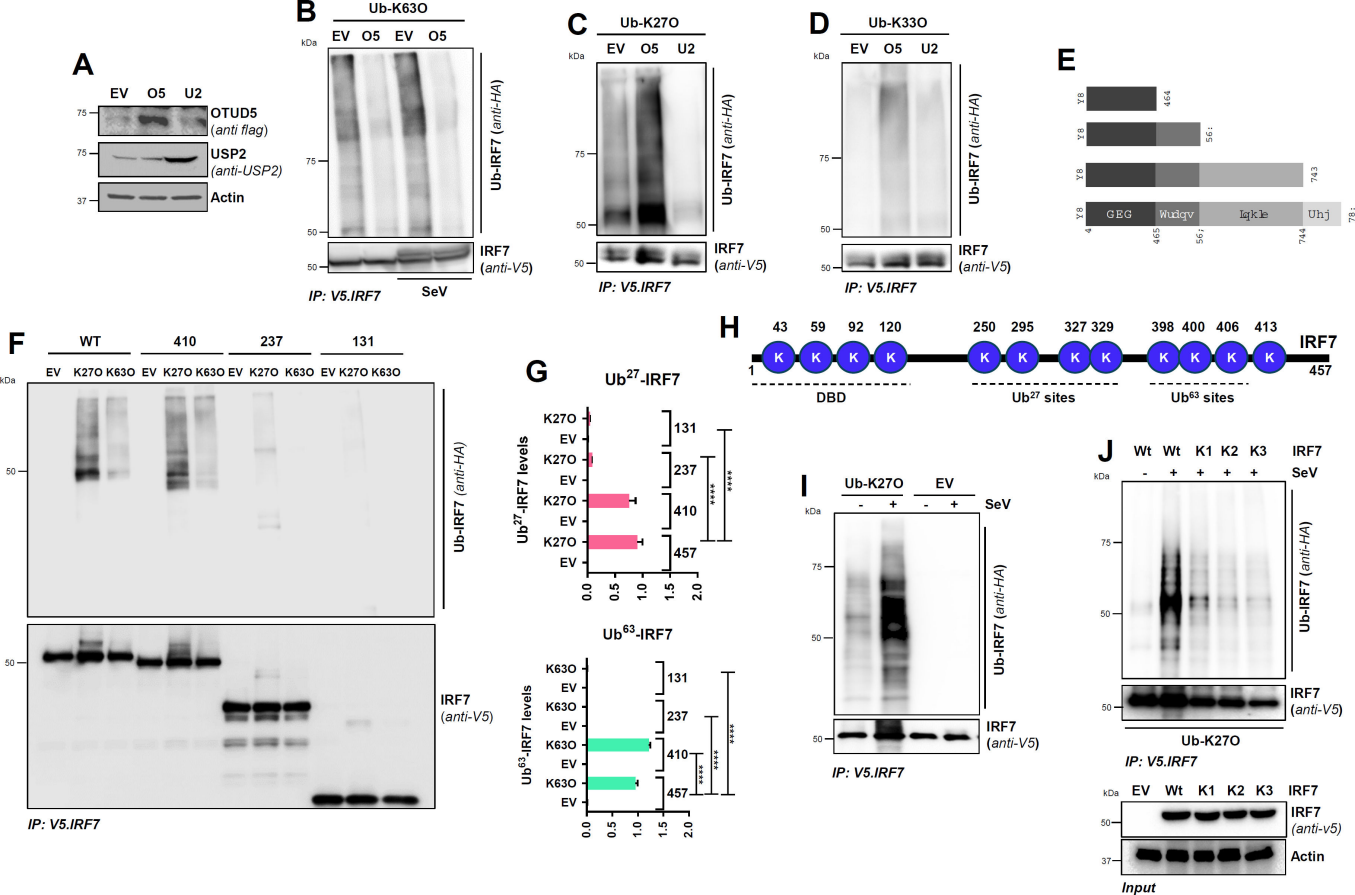
OTUD5 and USP2 regulate distinct ubiquitin linkages on IRF7. (**A**) HEK-3KO.IRF7 cells were transfected with Flag.OTUD5 or USP2 plasmids, and the cell lysates were analyzed by immunoblot, as indicated. (**B–D**) HEK-3KO.IRF7 cells were transfected with HA-tagged Ub-K63O, Ub-K27O, or Ub-K33O plasmids, in the absence or the presence of OTUD5 or USP2, as indicated, and infected with SeV. The cell lysates were immunoprecipitated with anti-V5 antibody and immunoblotted with anti-HA, as indicated. (**E–G**) Full-length and truncated IRF7 mutants (**E**) were co-transfected with Ub-K63O or Ub-K27O, followed by SeV infection. Ub-IRF7 levels were measured by IP and immunoblot (**F**) and quantified using ImageJ (**G**). (**H**) Schematic of IRF7 protein domains and putative ubiquitin linkage sites (K63 AND K27); DBD, DNA-binding domain. (**I**) Cells were transfected with HA.Ub-K27O and infected with SeV. Ub-IRF7 was analyzed as in panel **A**. (**J**) IRF7-WT or KR mutants (K1: K327R, K2: K329R, and K3: K327/329RR) were co-transfected with Ub-K27O, infected with SeV, and analyzed for Ub-IRF7. The lower panel shows the expression of the IRF7 mutants. EV, empty vector; Ub-IRF7, ubiquitinated IRF7.

To explore whether these interactions affect IRF7 ubiquitination, we examined the specificity of USP2 and OTUD5 for different ubiquitin linkages on IRF7 by ectopically expressing them in HEK-3KO.IRF7 cells (Fig. 6A). Since OTUD5 acts as a negative regulator of IRF7 activity, we first tested its effect on K63-linked ubiquitination, a modification known to activate IRF7. In HEK-3KO.IRF7 cells expressing the K63-only ubiquitin mutant (Ub-K63O), OTUD5 overexpression led to suppression of K63-linked ubiquitinated IRF7 (Ub^63^-IRF7), in both uninfected and SeV-infected conditions (Fig. 6B). Next, we tested the effects of USP2 and OTUD5 on K27- and K33-linked ubiquitination using Ub-K27O and Ub-K33O mutants. USP2, but not OTUD5, selectively reduced K27-linked ubiquitination of IRF7 (Ub^27^-IRF7), whereas neither enzyme affected Ub^33^-IRF7 (Fig. 6C and D). These results establish the linkage specificity of these DUBs: USP2 targets Ub^27^-IRF7 and OTUD5 targets Ub^63^-IRF7. To map the sites of these modifications, we used IRF7 deletion mutants and performed ubiquitination assays. Both Ub^27^- and Ub^63^-linked IRF7 levels were significantly reduced in the IRF7 (1–237) mutant compared to the IRF7 (1–410) construct (Fig. 6E through G), implicating the C-terminal region in ubiquitin modification. Based on our results from the IRF7 deletion mutants and prior knowledge of Ub^63^ sites, we postulated that lysines K327 and K329 are candidate Ub^27^ sites (Fig. 6H). We then generated IRF7 lysine-to-arginine mutants, K327R (K1), K329R (K2), and the double mutant KK327/329RR (K3), to assess their contributions to Ub^27^-IRF7 linkage (Fig. S1). SeV infection induced Ub^27^-IRF7 in HEK-3KO.IRF7 cells expressing Wt IRF7, while all three KR mutants exhibited significantly reduced Ub^27^-IRF7 levels (Fig. 6I and J). These results identify K327 and K329 as the key sites for K27-linked ubiquitination of IRF7 during viral infection and establish USP2 and OTUD5 as selective regulators of Ub^27^- and Ub^63^-linked IRF7, respectively.

### K27-linked ubiquitination inhibits IRF7 activation and transcriptional function

**Fig 7:**
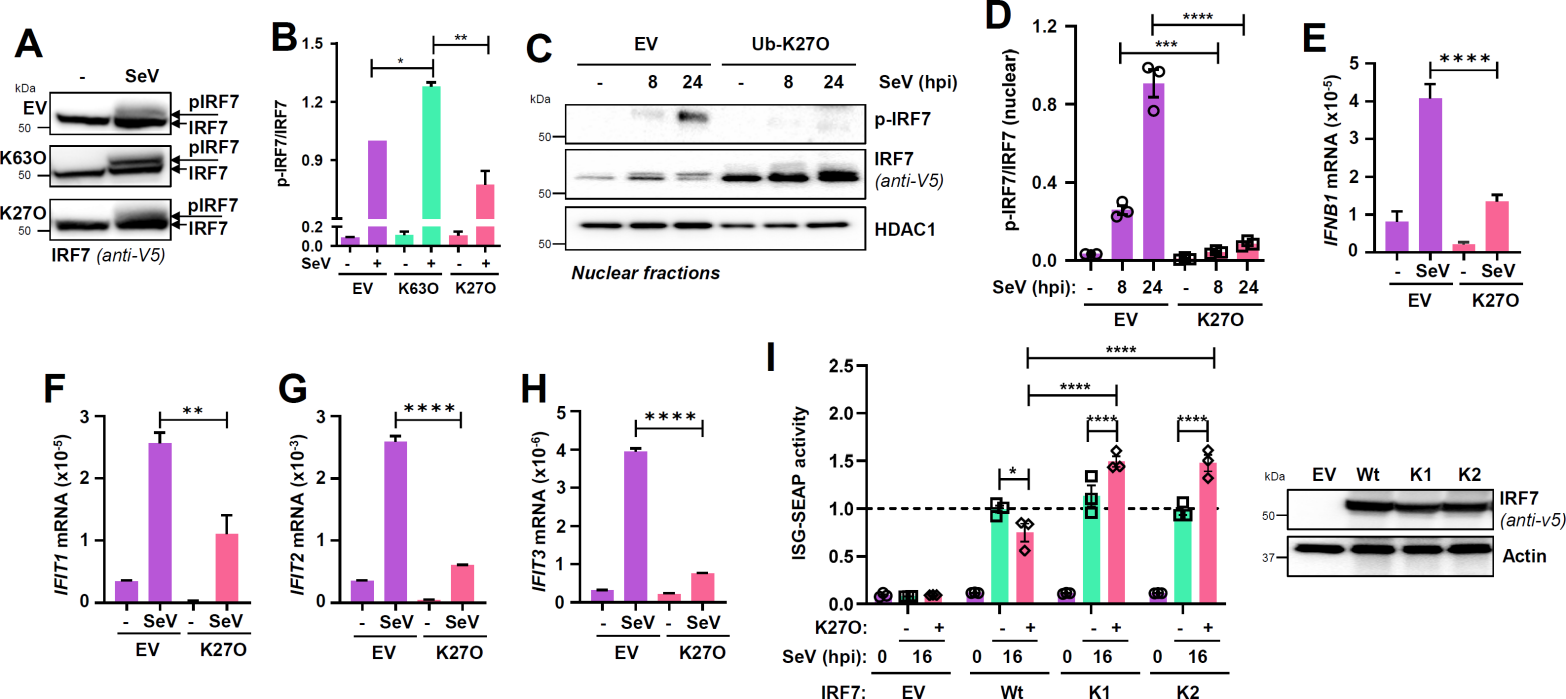
K27-linked ubiquitination suppresses IRF7 phosphorylation and transcriptional activity. (**A–B**) HEK-3KO.IRF7 cells were transfected with Ub-K63O or Ub-K27O plasmids and infected with SeV, and IRF7 phosphorylation (p-IRF7) was analyzed by immunoblot (**A**). Quantification of p-IRF7 and total IRF7 was performed using Image J (**B**). (**C–D**) Nuclear fractions from cells transfected with Ub-K27O and infected with SeV were analyzed for p-IRF7, total IRF7, and HDAC1. Nuclear p-IRF7 was quantified in panel **D**. (**E–H**) IRF7-WT and Ub-K27O were co-transfected in HEK-3KO cells, followed by SeV infection. Expression of *IFNB1*, *IFIT1*, *IFIT2*, and *IFIT3* was measured by qRT-PCR. (**I**) IRF3-deficient HEK293.ISG-SEAP (HEK.SEAP-3KO) cells were co-transfected with IRF7 (WT, K1, or K2) and Ub-K27O and infected with SeV, and SEAP activity was measured. IRF7 protein expression is shown in the right panel. EV, empty vector. The data represent mean ± SEM (**B and D-I**), *P < 0.05, **P < 0.001, ***P < 0.001, and ****P < 0.0001.

Given the novel Ub^27^-mediated regulation of IRF7 activity, we focused on dissecting the molecular mechanism. To investigate the functional impact of K27-linked ubiquitination on IRF7 activation, we analyzed the levels of phosphorylated IRF7 (p-IRF7) in HEK-3KO.IRF7 cells expressing either Ub-K27O or Ub-K63O. SDS-PAGE analysis revealed that Ub-K63O expression enhanced the lower mobility p-IRF7 band, while Ub-K27O suppressed it (Fig. 7A and B). We confirmed these findings by examining pSer437/438, key phosphorylation sites required for IRF7 activation, in nuclear extracts. Expression of Ub-K27O led to decreased pSer-IRF7 and accumulation of unphosphorylated IRF7 in the nucleus (Fig. 7C and D; Fig. S5), suggesting that K27-linked ubiquitination inhibits IRF7 phosphorylation. To directly assess transcriptional activity, we analyzed IRF7 target gene expression. Enrichment of Ub-K27O suppressed the induction of *IFNB1*, *IFIT1*, *IFIT2*, and *IFIT3* mRNAs (Fig. 7E through H), indicating that K27-linked ubiquitination impairs IRF7-driven antiviral gene expression. Finally, we evaluated the transcriptional activity of the K1 and K2 IRF7 mutants, which are defective in Ub^27^ modification. In the HEK-SEAP-3KO reporter cell line expressing ISRE-driven SEAP, both mutants exhibited significantly higher SEAP activity than Wt IRF7 (Fig. 7I). Notably, Ub-K27O suppressed SEAP activity in Wt IRF7-expressing cells but had no effect on K1 or K2 mutants (Fig. 7I), further demonstrating that K27-linked ubiquitination on K327 and K329 suppresses IRF7 transcriptional activity.

### USP2 is required for IRF7-induced gene expression following virus infection and TLR activation in primary macrophages

**Fig 8:**
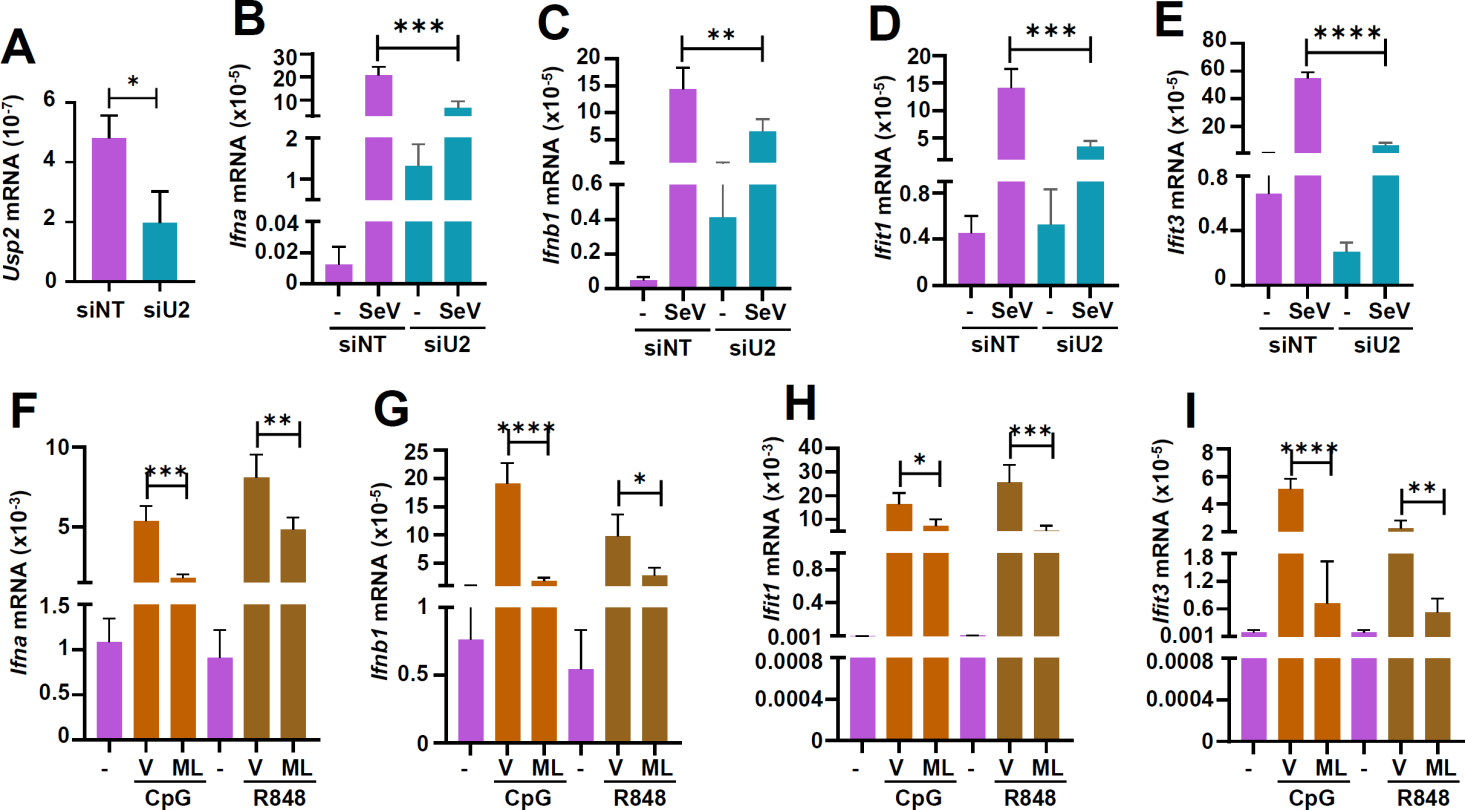
Genetic and pharmacological inhibition of USP2 suppress IRF7-target genes in primary BMDMs. (**A–E**) Primary BMDMs isolated from Wt C57BL/6 mice were transfected with non-targeting (siNT) or USP2-specific (siU2) siRNA and infected with SeV for 8h, when the mRNA levels of *Usp2*, *Ifna*, *Ifnb1*, *Ifit1*, and *Ifit3* were analyzed by qRT-PCR. (**F–I**) Primary BMDMs isolated from Wt C57BL/6 mice were pre-treated with ML364 (ML) for 1h and treated with CpG ODN (10 µg/mL) or R848 (10 µg/mL) for 8 h, when the mRNA levels of *Ifna*, *Ifnb1*, *Ifit1*, and *Ifit3* were analyzed by qRT-PCR. The data represent mean ± SEM, *P < 0.05, **P < 0.001, ***P < 0.001, and ****P < 0.0001.

To investigate the physiological relevance of USP2-mediated activation of IRF7, we used primary BMDMs and employed both genetic and pharmacological approaches. SiRNA-mediated knockdown of *Usp2* (Fig. 8A) significantly reduced the expression of IRF7 target genes, including *Ifna*, *Ifnb1*, *Ifit1*, and *Ifit3*, following SeV infection (Fig. 8B through E). To further assess the role of USP2 in IRF7 activation, we tested the effect of ML364, a selective USP2 inhibitor (29), on TLR7 and TLR9 signaling, both of which specifically activate IRF7 to induce antiviral genes. Pre-treatment with ML364 markedly inhibited the induction of *Ifna*, *Ifnb1*, *Ifit1*, and *Ifit3* upon stimulation with CpG ODN (a TLR9 agonist) and R848 (a TLR7 agonist; Fig. 8F through I). Together, these findings extend our cell line-based observations to primary macrophages and underscore the potential therapeutic value of targeting USP2 in regulating IRF7-driven antiviral responses.

## DISCUSSION

IRF7, a key member of the IRF family and the “master transcription factor” of type I IFNs, is predominantly expressed in immune and lung epithelial cells (8, 30). Its expression is further induced by viral infection or tissue damage. In resting cells, IRF7 resides in the cytosol in an inactive state. Activation of PRRs triggers IRF7 transcriptional activity, which is tightly regulated by post-translational modifications (7, 8). Phosphorylation of conserved serine residues (Ser425/426 or Ser437/438), primarily mediated by TBK1 or IKKε, leads to IRF7 nuclear translocation and gene induction. In addition to phosphorylation, ubiquitination plays a pivotal role in modulating IRF7 activity. K63-linked ubiquitination of IRF7, catalyzed by E3 ligases such as TRAF6 and NEURL3, promotes its transcriptional function (14, 31). However, ubiquitination is reversible and regulated by DUBs, which remove specific ubiquitin chains to maintain cellular homeostasis. The interplay between p-IRF7 and Ub-IRF7 remains incompletely understood. To dissect this regulatory axis, we conducted an unbiased genetic screen to identify IRF7-specific DUBs. Our screen uncovered two DUBs, OTUD5 and USP2, with opposing roles on IRF7 activation. OTUD5 functioned as a negative regulator, interacting with IRF7, removing K63-linked ubiquitin chains, and suppressing IRF7-dependent gene expression. In contrast, USP2 was required for IRF7 activity by deubiquitinating K27-linked IRF7, thereby facilitating transcriptional activation. Importantly, we found that K27-linked ubiquitination inhibits IRF7 phosphorylation, suggesting that K27-Ub acts as a negative regulatory linkage. Together, these findings suggest a model in which IRF7 recruits both inhibitory and activating DUBs to fine-tune its activity.

Mechanistically, our data suggest that IRF7 exists in a K27-linked ubiquitinated form under homeostatic conditions, maintaining the transcription factor in an inactive state. Upon stimulation, USP2 removes K27-linked chains, potentially allowing TRAF6 to catalyze activating K63-linked ubiquitination, thereby promoting IRF7 activity. Subsequently, OTUD5 may be recruited to remove K63-linked chains, thereby restoring IRF7 to an inactive state. Notably, OTUD5 recruitment occurs early in infection, possibly acting indirectly to reinforce K27-linkage. The identity of the E3 ligase responsible for K27-linked ubiquitination of IRF7 remains unknown. However, studies in zebrafish suggest that FBXO3 may catalyze this modification; FBXO3-deficient zebrafish are resistant to viral infection, supporting its role as a negative regulator of IRF7 (32). Whether FBXO3 serves a similar function in mammalian cells remains to be determined. Our findings also raise the possibility that mammalian IRF3 may undergo similar K27-linked regulation, given that FBXO3 modifies zebrafish IRF3 and that USP2 could serve as a shared regulatory DUB. These insights reveal a previously underappreciated noncanonical ubiquitin modification that negatively regulates IRF7 function. Further investigation is needed to determine whether K27-linked IRF7 plays roles in other physiological or pathological contexts, such as autoimmune diseases, where IRF7 is a critical contributor. Although K63-linked ubiquitination of IRF7 has been previously reported, most notably in EBV-infected cells, where LMP1 recruits TRAF6 to form a positive feedback loop involving IRF7 activation, our study demonstrates that this modification is subject to negative regulation by OTUD5 (14). Similarly, NEURL has recently been identified as a K63-specific E3 ligase promoting IRF7 antiviral function; NEURL-deficient mice exhibit increased viral susceptibility, underscoring the importance of K63-linked IRF7 in antiviral defense (31).

DUBs are emerging as central regulators of immune signaling. Their dysregulation can lead to immune dysfunction, inflammatory disease, and cancer (30, 33). However, the specific mechanisms by which DUBs are activated and recruited to IRF7 remain unclear. Prior studies, primarily in zebrafish, have implicated other DUBs, including USP8, which promotes autophagy-dependent degradation of IRF7 during SVCV infection, and A20, which suppresses IRF7 activity in EBV-infected cells (20, 34). To identify IRF7-specific regulators in human cells, we performed a siRNA-based screen in a system lacking IRF3, thereby isolating IRF7-driven responses. Although we characterized OTUD5 and USP2 in depth, other candidate DUBs identified in our screen (see Fig. 3F) may also regulate IRF7 and warrant further investigation. Our data indicate that OTUD5 expression is upregulated by viral infection or PRR stimulation, suggesting that it acts as a feedback inhibitor in antiviral signaling. We also observed increased IRF7-OTUD5 binding following SeV infection. In contrast, USP2 regulation is poorly understood; future studies are needed to clarify how its expression or activity is controlled. Notably, USP2 can be pharmacologically inhibited by ML364, a small molecule with reported antiviral and anticancer properties (29, 35). Our results indicate that ML364 inhibited TLR9- and TLR7-mediated activation of IRF7 (Fig. 8). This will be further investigated to examine its role in inhibiting IRF7’s pathogenic functions *in vivo*; however, several critical questions remain. What determines the specificity of IRF7-DUB interactions? In which cellular compartments do these interactions occur? How are DUBs temporally regulated during infection? Addressing these gaps will deepen our understanding of how IRF7 activity is dynamically regulated through ubiquitination. A previous study reported that OTUD5 (also known as DUBA), consistent with our findings, functions as a negative regulator of type I IFN production by targeting K63-linked ubiquitinated proteins (36). Specifically, OTUD5 was shown to deubiquitinate K63-ubiquitinated TRAF3, thereby suppressing its E3 ligase activity and attenuating downstream IFN signaling. In contrast, OTUD5 has also been described as a positive regulator of type I IFN responses through deubiquitination of K48-linked STING, thereby stabilizing the STING protein and enhancing signaling (37). Collectively, these studies indicate that OTUD5 can act on both K63- and K48-linked ubiquitin chains to fine-tune type I IFN responses in a context-dependent manner.

In summary, our study reveals that IRF7 activity is regulated by distinct ubiquitin linkages: K63-linked ubiquitination promotes transcriptional activation, whereas K27-linked ubiquitination inhibits it. Two DUBs, OTUD5 and USP2, selectively remove these linkages to suppress or enhance IRF7 activity, respectively. These findings reveal a new layer of IRF7 regulation and lay the groundwork for exploring DUBs as therapeutic targets in viral and autoimmune diseases where IRF7 plays a central role.

## Data Availability

All data presented in this paper are contained within the article.

## SUPPLEMENTAL MATERIAL

Supplemental Figures mbio.02820-25-s0001.pdf Figures S1–S5.

ASM does not own the copyrights to Supplemental Material that may be linked to, or accessed through, an article. The authors have granted ASM a non-exclusive, world-wide license to publish the Supplemental Material files. Please contact the corresponding author directly for reuse.
