# Supplementary material for: Opposing roles of deubiquitinases in the regulation of IRF7 transcriptional activity
Source: mBio. 2026 Jun 9;17(7):e02820-25. doi: 10.1128/mbio.02820-25 (PMC13343979; doi:10.1128/mbio.02820-25)
Supplement: Supplemental Figures — Figures S1–S5. [file mbio.02820-25-s0001.pdf]

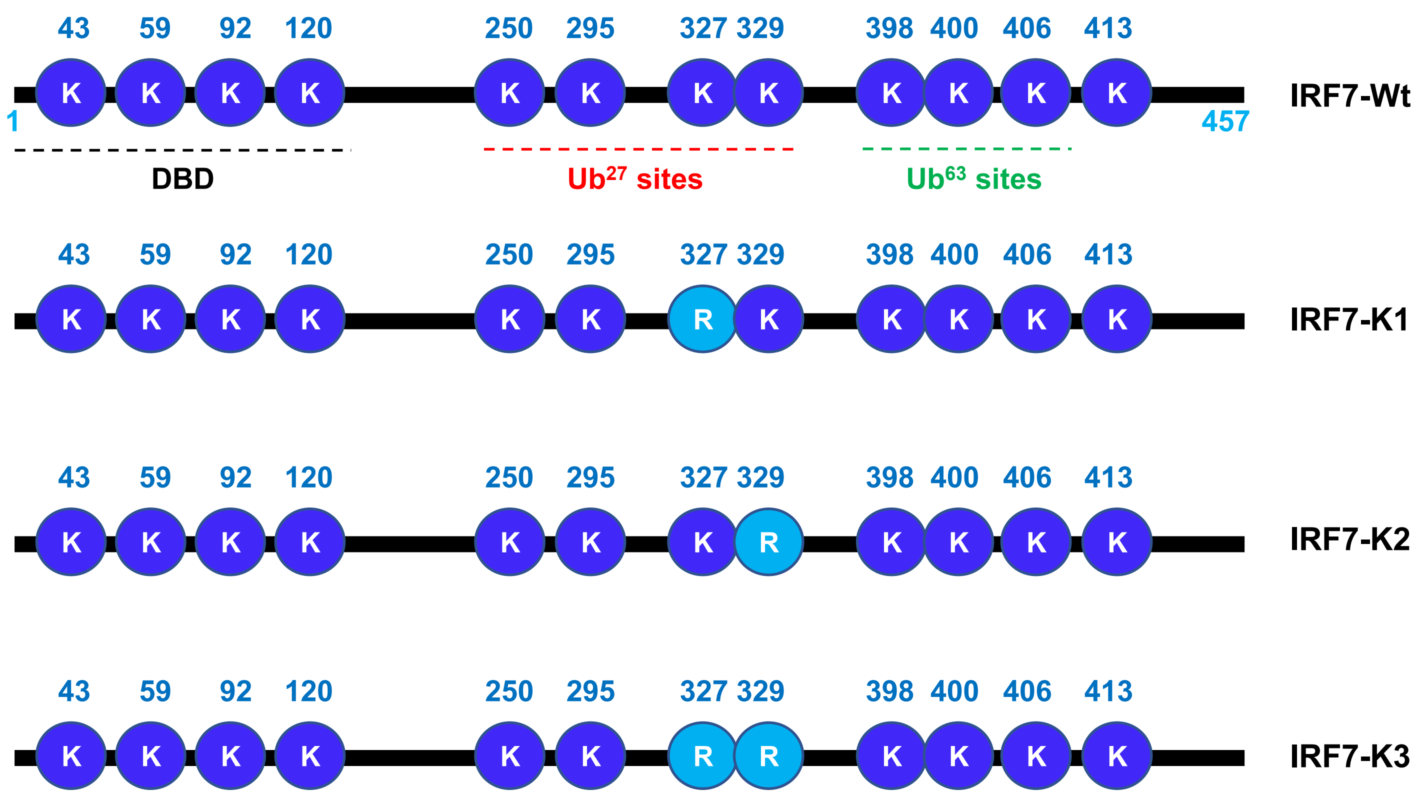

Fig S1. IRF7 and its lysine residues, and the mutants used in the study.

Gene name	Forward primer	Reverse primer
Ifnb1	CTTCTCCGTCATCTCCATAGGG	CACAGCCCTCTCCATCAACT
Ifna	CTTCCACAGGATCACTGTGTACCT	TTCTGCTCTGACCACCTCCC
Ifit1	CAGAAGCACACATTGAAGAA	TGTAAGTAGCCAGAGGAAGG
Ifit2	CGGAAAGCAGAGGAAATCAA	TGAAAGTTGCCATACCGAAG
Ifit3	GCCGTTACAGGGAAATACTGG	CCTCAACATCGGGGCTCT
Otud5	TACAACCGTCCTGTGGAGGTGT	TGGTAGCTGACACGGATGGGTT
Usp2	AGCCCATCTGAGTTCAAGACCC	GGTTCACCTCATTGTGGAGACC
IFNB1	CGCCGCATTGACCATCTA	GACATTAGCCAGGAGGTTCT
IFNA	GACTCCATCTTGGCTGTGA	TGATTTCTGCTCTGACAACCT
IFIT1	TCTCAGAGGAGCCTGGCTAAG	GTCACCAGACTCCTCACATTTGC
IFIT2	GGGAAACTATGCCTGGGTC	CCTTCGCTCTTTCATTTTGGTTTC
IFIT3	GAACATGCTGACCAAGCAGA	CAGTTGTGTCCACCCTTCCT
OTUD5	CAGGCTACAACAGTGAGGACGA	GAAGCCCTTCTTGTCTCGTAGG
USP2	GAGATACGCACCGCGCTTTGTT	GGTTGGACTTAGGTCTCAGTGTC
IAV-PR8-NP	TGCAAGGTTCAACTCTCCCT	GTTCCGGCTCTCTCTCACTT
18S	ATTGACGGAAGGGCACCACCAG	CAAATCGCTCCACCAACTAAGAACG

Fig S2. List of primers used for qRT-PCR analyses

ID	DUB
**A1**	OTUD5
**A2**	USP51
**A3**	PSMD14
**A4**	USP45
**A5**	PAN2
**A6**	USP30
**A7**	USP24
**A8**	USP35
**A9**	USP54
**A10**	USP40
**A11**	USP8
**A12**	JOSD2
**I1**	UEVLD
**I2**	ZRANB1
**I3**	USP34
**I4**	USP2
**I5**	USP36
**I6**	USP22
**I7**	USP31
**I8**	PRPF8
**I9**	UBL3
**I10**	CYLD
**I11**	USP39
**I12**	USP4

Fig S3. Activator and inhibitor DUB candidates selected for secondary screening.

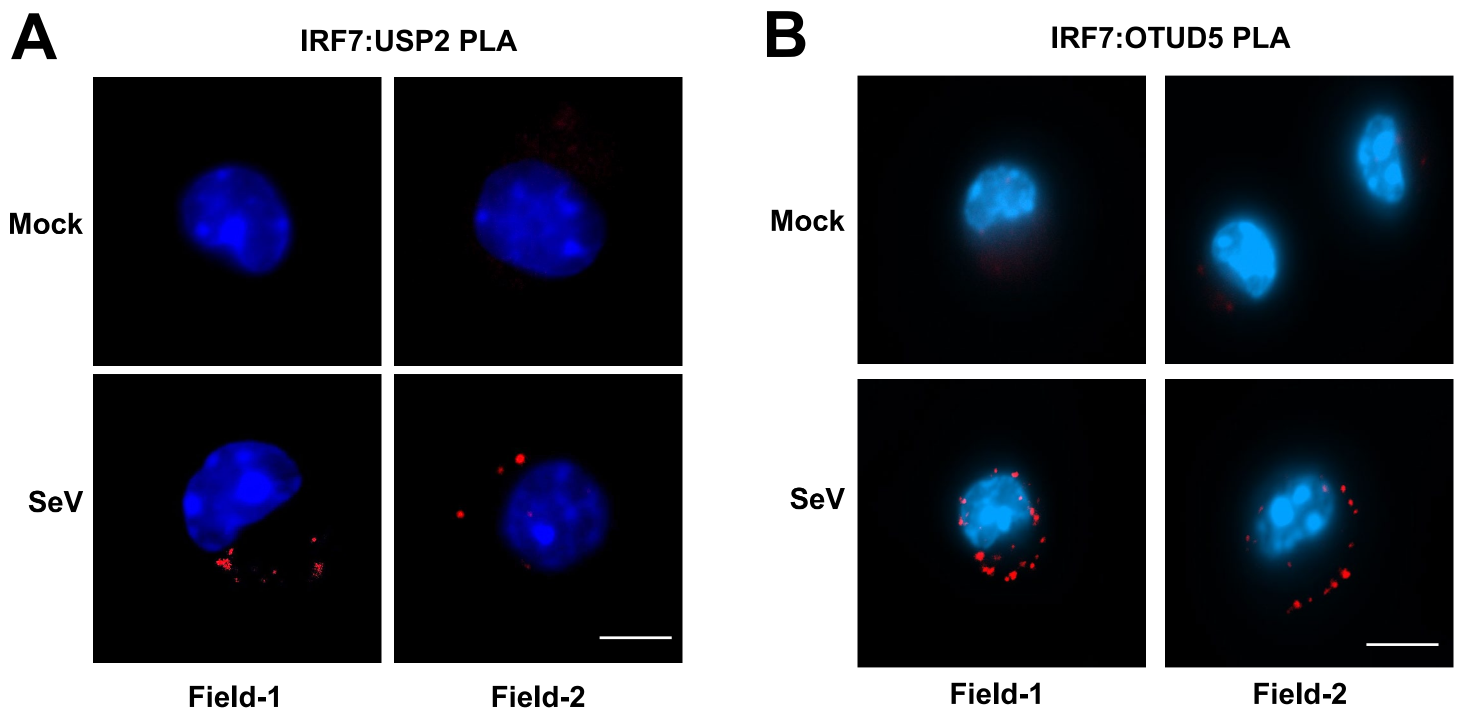

Fig S4. IRF7 interaction with DUBs in primary BMDMs. Wt BMDMs were either mock-infected or infected with SeV, and proximity ligation assay was performed using anti-IRF7 and anti-USP2 (A) or anti-IRF7 and anti-OTUD5 (B) antibodies.

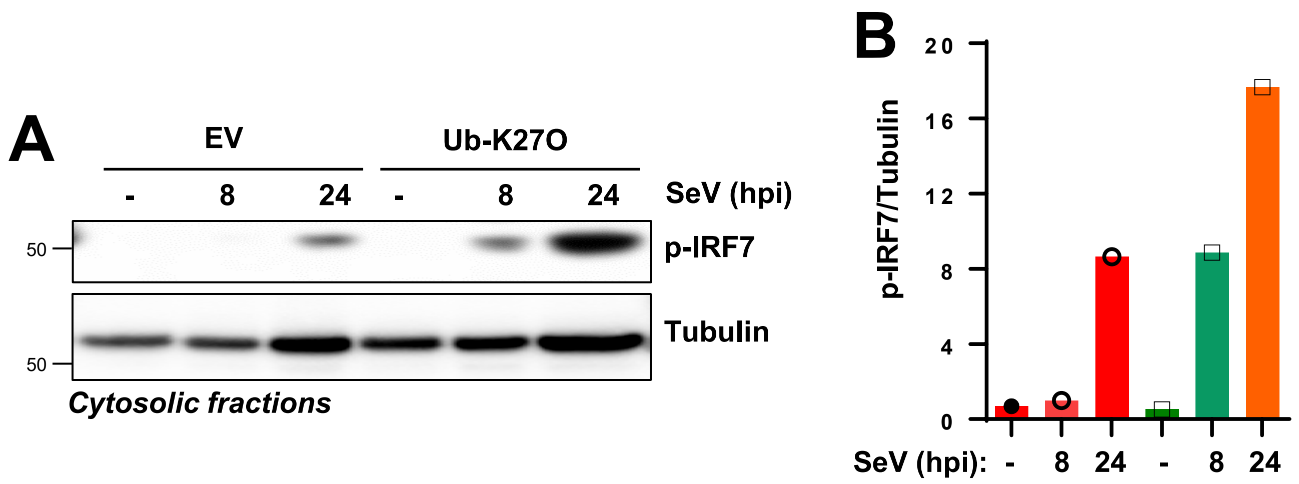

Fig S5. Ub[27] linkage leads to increased accumulation of p-IRF7 in the cytosol. KO.IRF7 cells were transfected with empty vector (EV) or Ub-K27O plasmids and infected with SeV, as indicated. The cytosolic fractions were analyzed for p-IRF7 and tubulin (A) and quantified using Image J (B).
